# A COMPARATIVE ANALYSIS ON HEALTHY LONGEVITY PHENOTYPES BETWEEN CHINESE AND ITALIAN CENTENARIANS

**DOI:** 10.1093/geroni/igz038.2242

**Published:** 2019-11-08

**Authors:** Huashuai Chen, Yi Zeng, Huashuai Chen, Yao Yao

**Affiliations:** 1 Duke University Medical Center, Durham, United States; 2 Medical School of Duke University, Durham, North Carolina, United States; 3 National School of Development, Peking University, Beijing, Beijing, China (People’s Republic)

## Abstract

This paper reviews and compares demographic, socioeconomic, behavioral (including diet) characteristics and heath phenotypes of centenarians in China and Italy. The results revealed that the interactions between familial longevity and any one of the three environmental factors (receipt of adequate medical care when ill as a child, number of living children, and household economic conditions) were significantly associated with the three health outcome indicators (IADL, self-rated life satisfaction, and anxiety-loneness) at old ages. We discovered that the effects of these environmental factors on the health outcome indicators were substantially stronger among elders who had no family history of longevity compared to centenarians’ children who likely carry genes and/or inherited healthy behavior and better lifestyle from long-lived parents.

